# P-150. Antibiogram Creation at King Faisal Hospital in Kigali, Rwanda to Inform Antimicrobial Stewardship Priorities

**DOI:** 10.1093/ofid/ofae631.355

**Published:** 2025-01-29

**Authors:** Jinan Sous, Michel Rafiki Gatera, Richard Nduwayezu, Mariska Kreuger, Jean-Pierre Uwizeyimana, Thierry Muvunyi, Dawd Siraj, Daniel Shirley

**Affiliations:** University of Wisconsin School of Medicine and Public Health, Madision, Wisconsin; King Faisal Hospital, Kigali, Kigali, Rwanda; King Faisal Hospital, Kigali, Kigali, Rwanda; King Faisal Hospital, Kigali, Kigali, Rwanda; King Faisal Hospital, Kigali, Kigali, Rwanda; King Faisal Hospital, Kigali, Kigali, Rwanda; University of Wisconsin School of Medicine and Public Health, Madision, Wisconsin; University of Wisconsin-Madison School of Medicine and Public Health, Madison, Wisconsin

## Abstract

**Background:**

Antimicrobial resistance (AMR) is a major global health challenge. King Faisal Hospital (KFH) in Kigali, Rwanda, is the country’s quaternary referral hospital. Creating antibiograms provides useful information for assessing the burden of resistance and for establishing pertinent antimicrobial use guidance.

**Figure 1:**
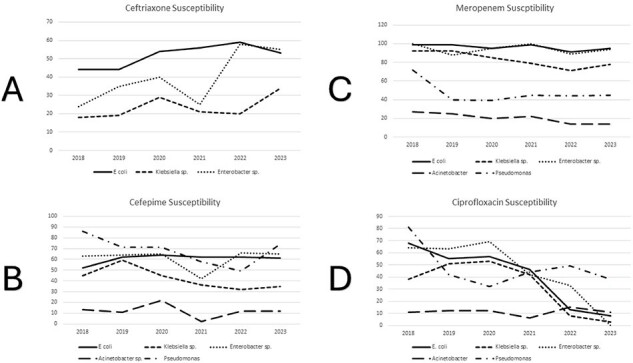
Susceptibility Patterns of Select Gram Negative Bacteria to Common Antibiotics

**Methods:**

We analyzed all positive cultures at KFH from January 1, 2018 – December 3, 2023. An electronic laboratory system was used to extract data. Duplicate samples were excluded based on CLSI guidelines. Data were organized by organism and susceptibility to reported antibiotics was recorded. Using this raw data, we created yearly antibiograms that showcased susceptibility patterns over time.

**Results:**

Susceptibilities of key Enterobacterales species were relatively low for ceftriaxone and cefepime but higher for piperacillin-tazobactam and meropenem; all were generally stable over time (Figure 1). Ciprofloxacin susceptibility for these organisms decreased over time. Methicillin (oxacillin) resistance in Staphylococcus aureus and vancomycin resistance in Enterococcus species were relatively rare and stable over time.

**Conclusion:**

Antimicrobial resistance is increasing worldwide and determining local resistance patterns through antibiograms is one important step to impacting this global problem. Decreased susceptibility to some antibacterial drugs was noted at KFH which helps inform areas for future study and intervention. Challenges to data collection were variations in lab reporting, data, and storage collection systems in the past five years. These findings also provide a basis for the creation of an antimicrobial stewardship program at KFH.

**Disclosures:**

**All Authors**: No reported disclosures

